# Biomarkers of benefit from cetuximab-based therapy in metastatic colorectal cancer: interaction of EGFR ligand expression with RAS/RAF, PIK3CA genotypes

**DOI:** 10.1186/1471-2407-13-49

**Published:** 2013-02-02

**Authors:** George Pentheroudakis, Vassiliki Kotoula, Wendy De Roock, George Kouvatseas, Pavlos Papakostas, Thomas Makatsoris, Demetris Papamichael, Ioannis Xanthakis, Joseph Sgouros, Despina Televantou, Georgia Kafiri, Athanassios C Tsamandas, Evangelia Razis, Eleni Galani, Dimitrios Bafaloukos, Ioannis Efstratiou, Iliada Bompolaki, Dimitrios Pectasides, Nicholas Pavlidis, Sabine Tejpar, George Fountzilas

**Affiliations:** 1 Department of Medical Oncology, Ioannina University Hospital, Ioannina, Greece; 2 Department of Pathology, Aristotle University of Thessaloniki School of Medicine, Thessaloniki, Greece; 3 Laboratory of Molecular Oncology, Hellenic Foundation for Cancer Research, Aristotle University of Thessaloniki School of Medicine, Thessaloniki, Greece; 4 Department of Oncology, Ziekenhuis Oost-Limburg, Genk, Belgium; 5 Health Data Specialists Ltd, Athens, Greece; 6 Department of Medical Oncology, “Hippokration” Hospital, Athens, Greece; 7 Division of Oncology, Department of Medicine, University Hospital, University of Patras Medical School, Patras, Greece; 8 Bank of Cyprus Oncology Center, Nicosia, Cyprus; 9 Department of Medical Oncology, “Papageorgiou” Hospital, Aristotle University of Thessaloniki School of Medicine, Thessaloniki, Greece; 10 Third Department of Medical Oncology, “Agii Anargiri” Cancer Hospital, Athens, Greece; 11 Department of Pathology, Ippokration Hospital, Athens, Greece; 12 Department of Pathology, University Hospital, University of Patras Medical School, Patras, Greece; 13 Third Department of Medical Oncology, “Hygeia” Hospital, Athens, Greece; 14 Second Department of Medical Oncology, “Metropolitan” Hospital, Piraeus, Greece; 15 First Department of Medical Oncology, “Metropolitan” Hospital, Piraeus, Greece; 16 Department of Pathology, “Papageorgiou” Hospital, Thessaloniki, Greece; 17 Oncology Department, General Hospital of Chania, Crete, Greece; 18 Oncology Section, Second Department of Internal Medicine, “Hippokration” Hospital, Athens, Greece

**Keywords:** Cetuximab, Epidermal growth factor receptor, EGFR ligands, KRAS, BRAF, PI3K gene mutations, Biomarkers

## Abstract

**Background:**

More than half of patients with KRAS-wild type advanced colorectal cancer (CRC) fail anti-EGFR monoclonal antibodies. We studied EGFR-axis messenger RNA (mRNA) expression and RAS, RAF, PIK3CA mutations in order to identify additional biomarkers of cetuximab efficacy.

**Methods:**

Previously genotyped (KRAS, NRAS, BRAF, PIK3CA mutations) formalin-fixed paraffin-embedded tumour biopsies of 226 cetuximab-treated CRC patients (1st to 3rd line therapy) were assessed for mRNA expression of epidermal growth factor receptor (EGFR) and its ligands EGF, Transofrming Growth Factor-a (TGFA), Amphiregulin (AREG) and Epiregulin (EREG) with real time quantitative PCR. Mutations were detected in 72 (31.9%) tumours for KRAS, in 6 (2.65%) for BRAF, in 7 (3.1%) for NRAS and in 37 (16.4%) for PIK3CA.

**Results:**

Only PIK3CA mutations occasionally coexisted with other gene mutations. In univariate analysis, prognostic significance for survival ( from metastases until death) was seen for BRAF mutations (Hazard Ratio HR 8.1, 95% CI 3.4-19), codon 12-only KRAS mutations (HR 1.62, 95% CI 1.1-2.4), high AREG mRNA expression only in KRAS wild type CRC (HR 0.47, 95% CI 0.3-0.7) and high EREG mRNA expression irrespective of KRAS mutation status (HR 0.45, 95% CI 0.28-0.7). EREG tumoural mRNA expression was significantly associated with a 2.26-fold increased likelihood of objective response to cetuximab therapy (RECIST 1.1). In multivariate analysis, favourable predictive factors were high AREG mRNA in KRAS wild type tumours, high EREG mRNA, low Ephrin A2 receptor mRNA. Cetuximab-treated patients with AREG-low KRAS wild type CRC fared very poorly, their survival being similar to KRAS mutant CRC. Patients with KRAS codon 13 or other non-codon 12 mutations had a median survival (30 months, 95% CI 20–35) similar to that of patients with KRAS wild-type (median survival 29 months, 95% CI 25–35), in contrast to patients with KRAS codon 12 mutations who fared worse (median survival 19 months, 95% CI 15–26).

**Conclusions:**

BRAF and codon 12 KRAS mutations predict for adverse outcome of CRC patients receiving cetuximab. AREG mRNA reflects EGFR signalling in KRAS wild type tumours, predicting for cetuximab efficacy when high and failure when low. EREG may have a prognostic role independent of KRAS mutation.

## Background

Colorectal cancer (CRC) is among the «big killers» in populations of developed societies, with a reported death toll of 50,000 yearly in the United States [1]. Recent advances in modern therapeutic strategies resulted in significant survival improvement of patients with metastatic disease. The Epidermal Growth Factor Receptor (EGFR) on the cancer cell surface relays signals of proliferation, angiogenesis, metastasis and antibodies binding it have been partly responsible for the observed outcomes improvement [2]. Cetuximab, a chimeric IgG1 monoclonal antibody (moAb) and panitumumab, a humanised IgG2 moAb are currently licensed for the treatment of patients with metastatic colorectal cancer either in combination with chemotherapy in the first and second line setting or as monotherapy for refractory disease. The need to identify tumours addicted to EGFR signalling and thus amenable to anti-EGFR therapeutic modulation became apparent early on, as response rates to cetuximab regimens in unselected patient populations were typically lower than 30% [3].

KRAS is a cytoplasmic GTP-binding protein with low inherent GTPase activity. When the KRAS protein is bound to GTP, it relays signals of cellular proliferation and inhibition of apoptosis, acting as a typical oncogene. KRAS mutations were observed mainly in gene exon 2, resulting in abrogated GTPase activity and locking the KRAS protein in the active KRAS-GTP conformation. By activating the RAS/RAF/MAPK axis downstream of EGFR, these mutations render therapeutic modulation of EGFR irrelevant [4]. Indeed, clinical data confirmed the predictive value of KRAS exon 2 mutations for resistance to cetuximab and panitumumab, leading to the license of these moAbs exclusively for the management of patients with KRAS-wild type colorectal cancers [5-7]. Despite application of such a «negative selection» biomarker, the KRAS-wild type patient population benefits from anti-EGFR strategies in less than half of cases. Research efforts towards identification of additional predictive biomarkers have generated interesting, though preliminary and at times conflicting data on the importance of tumour mRNA levels of EGFR ligands, of activating mutations in other genes such as BRAF, PIK3CA [8-11]. Finally, the codon localisation of KRAS mutations was found to possess differential transforming potential in cell cultures and to bear distinct predictive value for cetuximab resistance in clinical series [9].

We report a retrospective translational research project on previously diagnosed formalin-fixed paraffin-embedded (FFPE) colorectal carcinomas from 226 patients who were treated with cetuximab-based therapy in the first, second or third line setting for metastatic disease. In the context of a broad translational research protocol involving exploratory analyses of multiple biomarkes, our EGFR axis project aimed at screening for biomarkers of cetuximab benefit. It included studying gene expression of EGFR, its ligands epidermal growth factor (EGF), amphiregulin (AREG), epiregulin (EREG), transforming growth factor-a (TGFa) and the presence of activating mutations in KRAS, BRAF, NRAS, PIK3CA genes as well as the possible distinct effect of KRAS and PIK3CA mutations at various codons.

## Methods

### Patient selection

Two hundred and twenty-six patients with histologically established diagnosis of colorectal adenocarcinoma, for whom collection of tissue samples was part of their routine care and treatment, provided informed consent for the research use of their biologic material. The retrospective translational research protocol was approved by the Ethics Committee/Scientific Council of the General Hospital Papageorgiou, Thessaloniki (Meeting number 158/26-6-12). The study was performed in compliance with NCI-EORTC’s REMARK recommendations. Upon development of metastatic disease, these patients were managed with cetuximab-based therapy from May 2004 until December 2008 in Hellenic Cooperative Oncology Group (HeCOG)- affiliated centers. All FFPE blocks were retrospectively identified and quality-controlled by an experienced pathologist (Despina Televantou, DT) for the histological diagnosis of colorectal adenocarcinoma and for the assessment of tumour cell content. In case of less than 50% tumour cells in whole sections, manual tumour macrodissection was applied before further processing. Thus, all molecular samples contained molecular templates (DNA, RNA) above the 50% threshold.

### Tissue material and molecular studies

In total, 226 tissue blocks were available for DNA analysis. DNA samples had been centrally assessed for KRAS, NRAS, BRAF and PIK3CA mutations by using a Sequenom MALDI-TOF MassARRAY multiplex PCR method and the Sequenom MassARRAY Assay Design 3·1 software as previously published [8].

Corresponding sections or macrodissected tissue fragments from 199 available tissue blocks were lysed overnight and processed for RNA extraction with TRIZOL-LS (Invitrogen/Life Technologies, Paisley, UK), according to the manufacturers’ instructions. RNA was reverse transcribed with Superscript III and random hexamers (Invitrogen/Life Technologies). cDNAs were normalized at 25 ng/ul and stored at -20°C until use. mRNA expression was assessed with Taqman-MGB assays (Applied Biosystems/Life Technologies) for exon spanning amplicons, for the following targets with data in parentheses corresponding to assay ID, reference sequence, location and size of amplicon, in the same order: AREG (Hs00950669_m1, NM_001657.2, exons 3–4, 66 bp); EGF (Hs01099999_m1, NM_001963.3, exons 20–21, 70 bp); EGFR (Hs00193306_m1, NM_005228.3, exons 20–21, 69 bp); EREG (Hs00914313_m1, NM_001432.2, exons 3–4, 65 bp); and, TGFa (Hs00608187_m1, NM_001099691.1, NM_003236.2, exons 4–5, 70 bp).

Samples were run in duplicates in an ABI7900HT real time PCR system along with negative (no-template) and positive controls (commercially available reference RNA: TaqMan^®^ Control Total RNA, cat. no 4307281, Applied Biosystems/Life Technologies). As an endogenous control and for the normalization of CT (cycle threshold) values, an assay targeting GUSB mRNA (beta-glucuronidase [#4333767 F]) was used. GUSB was preferred over usually applied endogenous controls because (a) no pseudogenes have as yet been reported for this gene, and (b) it has been identified as one among the best preserved mRNA targets in FFPE tissues [12]. To obtain linear Relative Quantification (RQ) values, relative expression was assessed as (40-dCT), as previously described, whereby dCT (or deltaCT) was calculated as (average target CT) – (average GUSB CT) from all eligible measurements [13]. Samples were considered eligible for analysis when both GUSB CTs in duplicates were <36 and when duplicate dRQ values (dRQ1 – dRQ2) were <0.85. As observed upon consecutive runs of 30 eligible samples (15% of all samples), minimum-maximum deltaRQs for all assays were <1.5 (AREG: 0.43; EGF: 0.85; EGFR: 1.2; EREG: 0.69; TGFa: 0.64). RNA studies were performed in the Laboratory of Molecular Oncology, Aristotle University of Thessaloniki, Greece.

### Endpoints

The study endpoints were a) Survival, from the time of diagnosis of metastatic disease until death or last follow-up, b) Best objective response rate (ORR) to any line of cetuximab-based therapy by RECIST 1.1 criteria [14]. A progression-free survival endpoint was not used intentionally because in the setting of cetuximab use in various lines of therapy, it could be influenced by the distribution of cetuximab therapy line in various categories of biomarkers under study.

We analysed the prognostic/predictive impact of different types of mutations of KRAS (codon 12 versus codon 13 versus other mutations versus wild type) and PIK3CA genes (exon 9 versus exon 20 versus wild-type). We also compared outcomes of cases with complex mutational phenotypes for prognostic/predictive utility. Complex Genotype comparison 1 (CG1) compared outcomes of KRAS wild type versus KRAS mutant versus [PIK3CA mutant + (KRAS or BRAF mutant)]. CG comparison 2 (CG2) compared outcomes of PIK3CA wild type versus PIK3CA mutant versus [PIK3CA mutant + (KRAS or BRAF mutant)], while CG comparison 3 (CG3) outcomes of all KRAS, BRAF, PIK3CA wild type versus any of them mutant versus [PIK3CA mutant + (KRAS or BRAF mutant)].

### Statistical analysis

Cut-offs for each mRNA marker were selected either as natural breaks in the distributional profiles of the corresponding RQ values or, if these were not apparent, as RQ value quartiles, which were examined for statistically significant associations with survival at the 5% significance level. If a cut-off was statistically significant it was used to classify low and high expressing tumours. In the absence of a candidate cut-off, mRNA markers were examined as continuous variables. The criterion for selecting cut-offs was the minimum p-value method. In cases of more than one plausible candidate points, graphical diagnostic plots using martingale residuals were used for time to event endpoints and the method of Miller and Seigmund for ORR [15]. Time-to-event distributions were estimated using Kaplan-Meier curves. Associations between biomarkers, mutations and basic patient and tumour characteristics were examined using the Fisher’s exact test or the Chi-Square test. Cox proportional hazards and logistic regression were used for univariate survival and ORR analyses respectively. Significant correlations between parameters were tested as interactions for prognostic significance in terms of survival and ORR. The multivariate model contained every significant variable and interaction from univariate analyses. A backward selection procedure with 15% removal cut-off was used.

The major aim of our research proposal is the characterization of molecular biomarkers which would help to predict survival to cetuximab therapy. The study’s power justification scheme was based upon the following assumptions: a) expected follow up of 60 months, based on 56 months accrual duration with 2 years additional follow-up period b) ratio of expressed to non-expressed samples of either 1:3, 1:1 or 3:1 and c) HR of 0.4-0.5. With this background information and risk assumptions the sample of 226 patients provided power from 91% to 99% at the 5% level of significance for performing a two sided log-rank test.

## Results

### Patient and tumour demographics

A total of 226 patients of a median age of 62.6 years (range 26–85) underwent excisional or incisional biopsy of colorectal adenocarcinoma, of whom 83 (36.8%) were diagnosed with localised (stage I-III) colon cancer and 137 (60.6%) with metastatic disease (Table 1). The primary tumour was located in the left colon (distal transverse to rectum) in 165 (73.0%) of cases and in the right colon (caecum to proximal transverse) in 60 (26.6%). Rectal tumours accounted for 71 cases (31%).

**Table 1:** Disease demographics and management data

**Parameter**	**N (%)**
*Age (median)*	62.6 years
*Gender (Female/Male)*	97 (43.0%)/129 (57.0%)
*Histological Grade*	
1–2	170 (75.2%)
3-4	46 (20.4%)
*Obstruction Yes/No*	30 (13.2%)/179 (79.2%)
*Perforation Yes/No*	9 (4.0%)/201 (89.0%)
*Primary Site Left/Right*	165 (73.0%)/60 (26.6%)
Rectum	71 (31.0%)
*TNM stage at biopsy*	
I-III versus IV	83 (36.8%)/137 (60.6%)
*Median Follow Up*	73.6 months
*Deaths*	175 (77.4%)
*Line of therapy at Cetuximab administration*	
1st line	38 (16.8%)
2nd line	108 (47.8%)
3rd line and beyond	80 (35.4%)
*Type of therapy at Cetuximab administration*	
Irinotecan-based	153 (48.7%)
Oxaliplatin-based	84 (26.7%)
Both irinotecan and oxaliplatin	29 (9.2%)
Only fluoropyrimidine	6 (1.9%)
Single-agent Cetuximab	42 (13.4%)
*Objective Response to Cetuximab*	
*All lines of therapy*	
Complete Respone (CR)	2 (0.9%)
Partial Response (PR)	55 (24.4%)
ORR (CR+PR)	57 (25.3%)
Stable disease (SD)	66 (29.3%)
Progressive disease (PD)	70 (31.1%)
*ORR by line of Cetuximab therapy*	
1st line	17 (44.7%)
2nd line	23 (21.5%)
3rd line	14 (22.9%)

From May 2004 until December 2008, cetuximab had been administered as an intravenous infusion according to standard regimens (loading dose 400 mg/m2 followed by weekly 250 mg/m2) as monotherapy (42, 13.4%) or with regimes based on irinotecan (153, 48.7%), oxaliplatin (84, 26.7%) or both agents (29, 9.2%). The lines of therapy during which cetuximab was administered were 1st line in 38 (16.8%), 2nd line in 108 (47.8%), 3rd and beyond in 80 (35.4%) (Table 1). Of note, as the period of therapy extended from 2004 until December 2008, an unselected patient population received cetuximab-based therapies, irrespective of KRAS mutational status.

### mRNA markers and somatic genotypes

Out of the 199 samples tested for mRNA expression, three were found not eligible for all targets. In total, 84.4% of samples were eligible for AREG analysis; 83.9% for EGF; 84.4% for EGFR; 80.4% for EREG; and 81.4% for TGFa. Out of the 226 DNA samples screened for the presence of mutations, genotyping was successful in 205 samples for KRAS (90.8%), 159 (70.4%) for NRAS, 220 for BRAF and 220 for PIK3CA (97.3% in each case) (Table 2). KRAS mutations were detected in 72 tumours (31.9%), mostly in exon 2, in codon 12 in 45 cases (20.0%) and in codon 13 in 16 cases (7.0%), while BRAF V600E mutations were found in 6 cases (2.6%). NRAS mutations were also quite rare, found only in 7 cases (3.1%). PIK3CA mutations were detected in 37 tumours (16.4%), 20 in exon 9 (8.8%) and only five in exon 20 (2.2%). This genotypic analysis was included in a previous publication [8].

**Table 2:** mRNA biomarkers and gene mutation status

**Biomarkers and mutations**	**N (%)**	**Median RQ**	**Interquartile range (IQR)**
**AREG**	168	39.9	38.7-40.9
**EREG**	160	36.4	34.7-37.9
**EGF**	167	29.3	27.9-33.6
**TGFa**	162	37.1	36.2-37.9
**EGFR**	168	38.3	37.7-39.2
**KRAS N=205**			
*All mutations*	72 (31.9%)		
*G12*	45 (20.0%)		
*G13*	16 (7.0%)		
*Q61*	4 (1.7%)		
*A146*	7 (3.1%)		
*Wild type*	133 (58.8%)		
*Missing*	21 (9.3%)		
**NRAS N=159**			
*All mutations*	7 (3.1%)		
*Q61*	5 (2.2%)		
*G12*	2 (0.9%)		
*Wild type*	152 (67.3%)		
*Missing*	67 (29.6%)		
**BRAF N=220**			
*V600E*	6 (2.6%)		
*Wild type*	214 (94.7%)		
*Missing*	6 (2.6%)		
**PIK3CA N=220**			
*All mutations*	37 (16.4%)		
*Exon 9*	20 (8.8%)		
*Exon 20*	5 (2.2%)		
*Other*	12 (5.3%)		
*Wild type*	183 (81.0%)		
*Missing*	6 (2.6%)		

Correlation analysis revealed that mutations in KRAS, BRAF, NRAS genes were mutually exclusive in all evaluable cases. On the contrary, mutations in the PIK3CA gene co-existed with KRAS mutations in 17 cases and with NRAS mutations in two. Statistically significant associations at the 2-sided p<0.05 level were seen between wild type status of KRAS, BRAF genes and high AREG, EREG, EGFR mRNA levels. Among KRAS-wild type cases, 81.4% were seen in AREG-high and 57.4% in EREG-high tumours, while of KRAS-mutated cases only 63.9% were observed in AREG-high and only 35.7% in EREG-high tumours. Similarly, among BRAF-wild type cases, 76.1% were seen in AREG-high and 50.7% in EREG-high tumours, while of BRAF-mutated cases only two out of six (33.3%) were observed in AREG-high and none in EREG-high tumours. Regarding the primary location, 64.8% of KRAS mutations (p=0.07) and 58% of PIK3CA mutations (p=0.036) occurred in left-sided colorectal tumours, whereas four (66.7%) of BRAF mutations in right-sided colon carcinomas (p=0.024). mRNA RQ values were examined as continuous variables and significant correlations at Spearmann’s R>0.4 were found between AREG and EREG mRNA (R=0.63, 95% CI 0.53-0.72, p=0.0005).

### Factors with predictive significance at univariate analysis

At a median follow-up of 73.6 months, 215 patients (95.1%) had experienced disease progression and 163 (93.1%) had died. The median survival was was 27 months (95% CI 25–31). At univariate analysis, in the present cohort of patients treated with cetuximab the presence of BRAF mutations was significantly associated with an 8.1-fold increased risk of death compared to patients harbouring BRAF-wild type tumours. When all types of mutations were pooled for each gene, KRAS (HR 1.28, p=0.14), NRAS (HR 1.43, p=0.36) and PIK3CA (HR 1.27, p=0.25) mutations did not have prognostic/predictive utility. However, when types of mutations were analysed separately for each gene, KRAS codon 12 mutations were predictive for increased risk of death (HR 1.62, p=0.014).

Among parameters studied for gene expression, only the 25th percentile of AREG mRNA values and the 75th percentile of EREG mRNA values dichotomized cases into groups with different outcome and were selected as optimal cut-offs. None of EGF, TGFa, EGFR mRNA levels disclosed cut-off points of prognostic significance. Cetuximab therapy in patients harbouring AREG-high (HR 0.47, p=0.0002) or EREG-high (HR 0.45, p=0.0009) tumours resulted in reduced risk of death. EGFR, EGF, TGFa mRNA expression had no significant prognostic/predictive value, either in the entire cohort or in the KRAS-wild type versus KRAS mutant cases.

**Figure 1:**
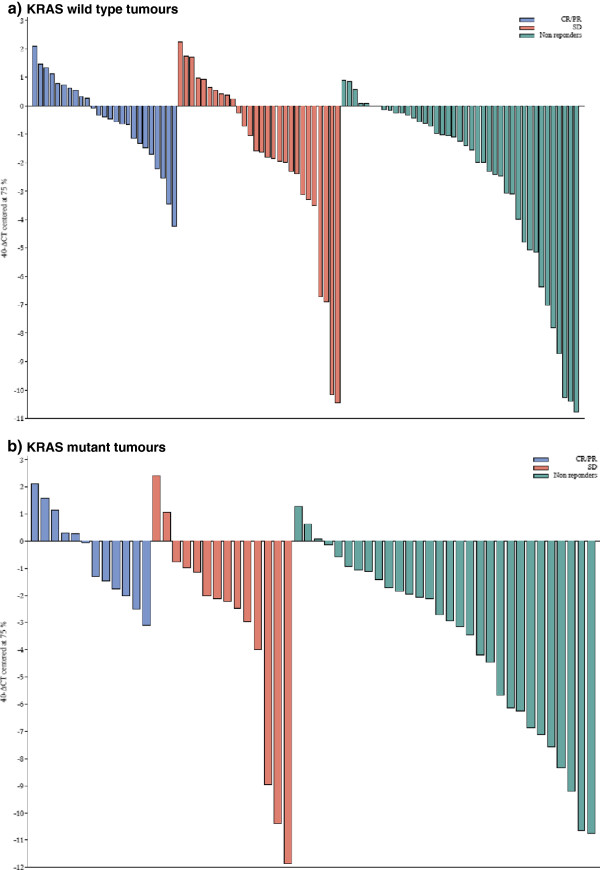
**Waterfall plot of tumoural EREG mRNA levels in responding and non-responding patients by KRAS mutation status. a)** KRAS wild type tumours, **b)** KRAS mutant tumours. X-axis corresponds to the 75th percentile EREG mRNA RQ values.

Regarding objective tumour response to any line of cetuximab-based therapy, tumoural AREG and EREG mRNA expression levels were associated with tumour shrinkage as continuous variables. AREG was not statistically significant as categorical variable but EREG mRNA at the 75th percentile was associated with a 2.26-fold increased likelihood of tumour response to cetuximab compared to tumours with lower EREG mRNA expression. AREG failed to predict response in either KRAS wild type or mutated cases. The expression levels of EREG in the tumours of responding and non-responding patients by KRAS gene status is depicted in a waterfall plot (Figure 1). Of note, in an exploratory analysis, the predictive significance for objective response was maintained only in KRAS mutated CRC, in which EREG had an odds ratio for response of 5.4 (95% CI 1.2-23.8, p=0.024). By contrast, in KRAS-wild type tumours EREG failed to significantly predict for response to therapy (OR 2.2, 95% CI 0.83-5.86, p=0.11). EGF, TGFa and EGFR mRNA expression had no predictive utility for response to cetuximab in the whole cohort, neither in KRAS wild-type or KRAS mutated cases. All factors with potential predictive utility for cetuximab benefit are summarized in Table 3.

**Table 3:** Prognostic/predictive factors at univariate analysis

**Predictive for survival**	**N**	**HR**	**95% CI**	**Median survival (months)**	**2-sided P value**
**AREG mRNA (25th percentile)**		0.47	0.31-0.70		0.0002
High	124			29	
Low	40			16	
**EREG mRNA (75th percentile)**		0.45	0.28-0.72		0.0009
High	39			36	
Low	117			23	
**BRAF**		8.1	3.44-19.0		0.00005
Mutation	6			12	
Wild type	208			28	
**KRAS codon 12**		1.62	1.10-2.38		0.014
Mutation	44			19	
Wild type	129			29	
**Predictive for Response**		**Odds Ratio for Response**	**95% CI**		**2-sided P**
**AREG mRNA at 25th percentile**		1.59	0.67-3.77		0.29
High vs. Low					
**EREG mRNA at 75th percentile**		2.26	1.04-4.91		0.04
High vs. Low					

### Interactions and factors with predictive utility at multivariate analysis

**Figure 2:**
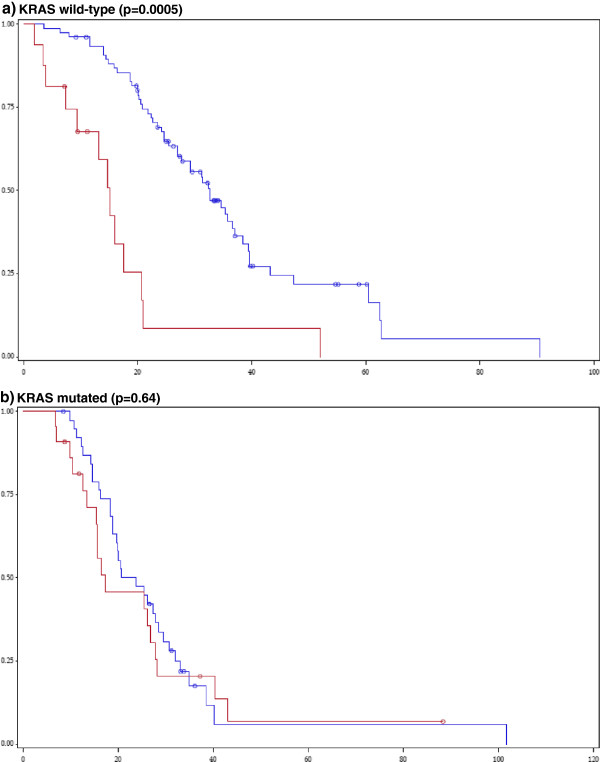
**Impact of AREG mRNA levels on cetuximab-treated patient survival by KRAS tumour mutation.** (Blue line: AREG-high, Red line: AREG-low). **a)** KRAS wild-type (p=0.0005), **b)** KRAS mutated (p=0.64).

**Figure 3:**
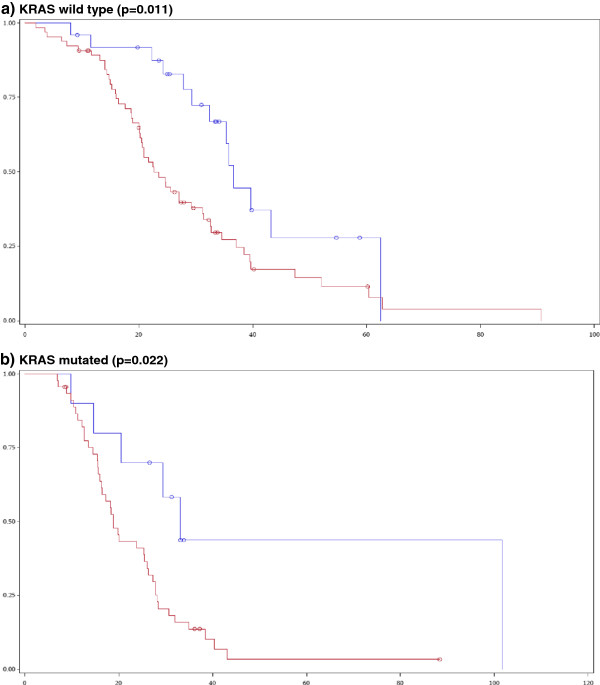
**Impact of EREG mRNA levels on cetuximab-treated patient survival by KRAS tumour mutation.** (Blue line: EREG-high, Red line: EREG-low).**a)** KRAS wild type (p=0.011), **b)** KRAS mutated (p=0.022).

Significant interactions (at the 1% significance level) between study variables for prognostic/predictive utility (survival, ORR) were sought. In terms of impact on survival, significant interactions were found between AREG mRNA levels and mutational status of the KRAS gene (p=0.0033). High mRNA levels of AREG predicted for longer survival in patients with KRAS wild-type (median survival 33 vs. 15 months, p=0.0005, Figure 2), but not in KRAS mutant tumours (median survival 22 vs. 17 months, p=0.64). In contrast, high mRNA levels of EREG were associated with favourable prognosis irrespective of KRAS mutational status. In KRAS wild type cases, the median survival was 37 months for EREG-high as compared to 23 months for EREG-low expressing tumours (p=0.01), while in KRAS mutated tumours median survival was 33 versus 19 months (p=0.02) in EREG-high vs. low cases (Figure 3). Similar findings for EREG high vs. low were seen when we examined complex cancer genotypes: a) KRAS+BRAF, both wild type vs. any mutant, b) KRAS+BRAF+PIK3CA+NRAS, all wild type vs. any mutant. AREG mRNA had a favourable predictive significance in wild type cases only, whereas EREG mRNA preserved its significance in wild type as well as in mutant cases.

A multivariate analysis was performed in the context of a broader mRNA profiling project of several biomarkers (IGFBP2, IGF1R, cMET, EGFR, TGFa, EphA2, HER2, HER3, HER4, Table 4). In the presence of wild-type KRAS, high AREG mRNA levels were independently associated with 83.0% reduction in the risk of death compared to patients with AREG-low tumours. High EREG tumoural mRNA was a favourable outcome predictor for cetuximab-treated patients, irrespective of KRAS mutation status. High Ephrin A2 (EphA2) mRNA and advanced age were adverse independent prognosticators. Finally, among cetuximab-treated patients with AREG-low tumours, those with mutant KRAS fared significantly better than patients harbouring colon cancer with KRAS wild type (81% decreased risk of death).

**Table 4:** Prognostic/predictive factors at multivariate analysis

**Survival (N=136)**	**HR**	**95% CI**	**Wald-p**
**Age**			
> median	1.64	1.08-2.48	0.0199
**EPHA2**			
High vs. Low	1.67	1.05-2.63	0.0287
**EREG**			
High vs. Low	0.38	0.21-0.66	0.0006
**AREG/KRAS interaction**	7.98	2.98-21.40	<.0001
AREG high in KRAS mutant vs			
AREG high in KRAS wild type			
**AREG**			
AREG high vs. low for KRAS wild type	0.17	0.08-0.34	<.0001
AREG high vs. low for KRAS mutant	1.33	0.67-2.61	0.4165
**KRAS**			
KRAS mutant vs. wild type for AREG high	1.49	0.90-2.45	0.1184
KRAS mutant vs. wild type for AREG low	0.19	0. 08–0.43	<.0001

### Impact of distinct types of KRAS and PIK3CA mutations on outcome of cetuximab-treated patients

**Figure 4:**
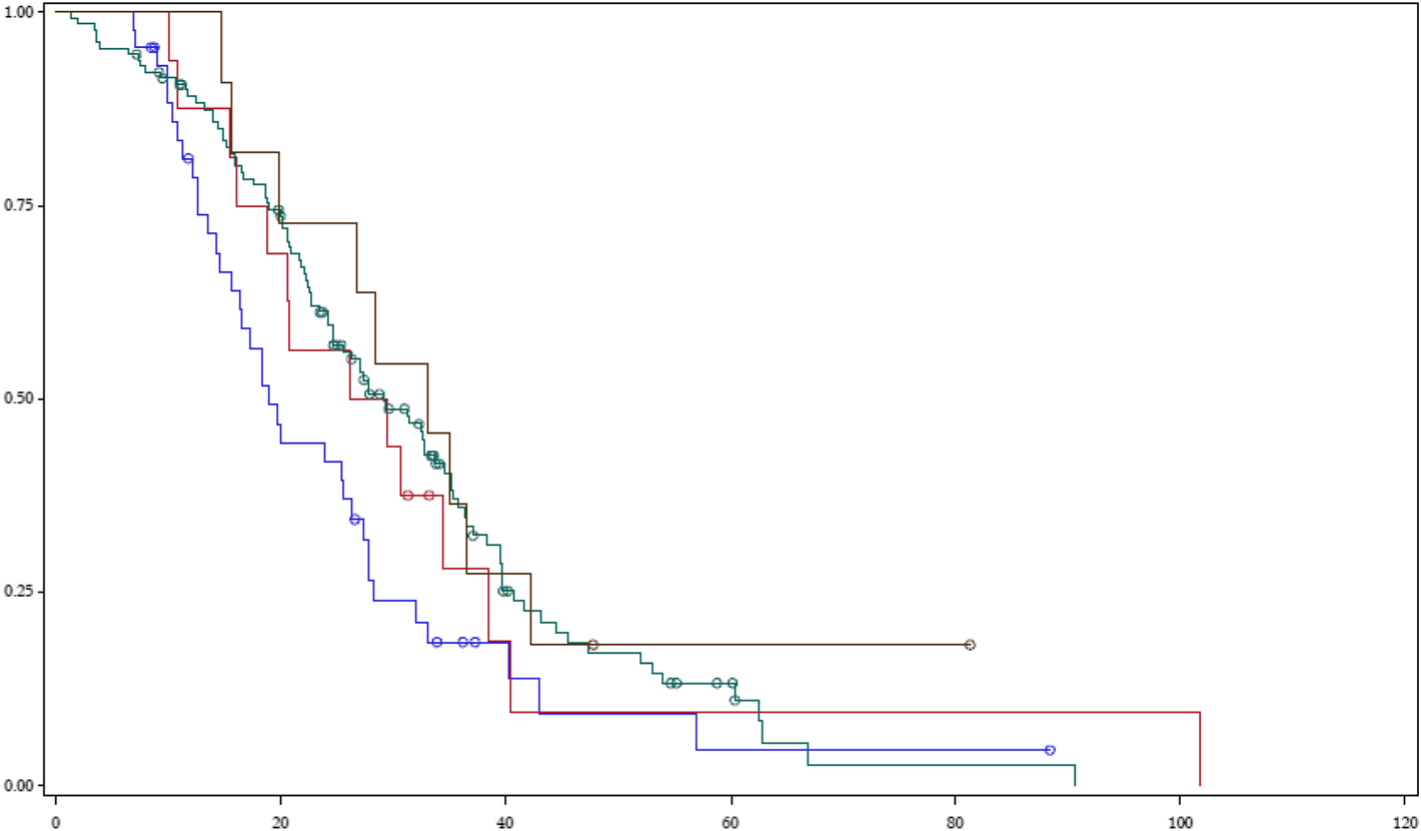
**Survival of cetuximab-treated patients by type of KRAS mutation in tumour.** Blue line: codon 12 mutations, Red line: codon 13 mutations, Green line: wild-type, Purple line: other KRAS mutations.

We examined different types of KRAS and PIK3CA mutations for potentially distinct impact on patient survival. Regarding KRAS, cetuximab-treated patients with codon 12 mutations had a median survival of 19 months (95% CI 15–26), significantly lower (p=0.033) than the median survival of patients with other KRAS mutations (30 months, 95% CI 20–35) and that of patients with wild-type KRAS (29 months, 95% CI 25–35). Further subgroup analyses showed that the median survival in patients with codon 12 KRAS mutations was also lower than the median survival of all other patient subgroups (other KRAS mutations or KRAS wild-type). Specifically, patients with codon 13 mutations reached a median survival of 28 months (95% CI 16–39), those with other rarer KRAS mutations a median survival of 33 months (95% CI 16–42) and patients with KRAS wild-type tumours a median survival of 29 months (95% CI 25–35). Despite the rather small size of compared subgroups, a trend for statistical significance was evident (p=0.068, Figure 4). No difference in patient outcomes was found when we compared tumours with PIK3CA exon 9 versus exon 20 mutations versus PIK3CA wild-type status, as survival times clustered from 25 to 29 months (p=0.31). The only complex genotype that harboured significance for cetuximab benefit was CG3 (p=0.019): Patients with tumours wild-type for all PIK3CA, KRAS, BRAF reached a median survival of 32 months (95% CI 25–36), those with a PIK3CA mutation along with KRAS or BRAF mutation had a median survival of 26 months (95% CI 16–32) while patients with any single mutation in KRAS, BRAF or PIK3CA genes had a median survival of 22 months (95% CI 19–28). Apparently, concurrent mutations in PIK3CA along with KRAS or BRAF mutations carried rather weak additional adverse prediction.

## Discussion

Despite exclusion of 30-40% of patients with KRAS-mutant tumours, cetuximab-based regimens fail in more than half of patients bearing KRAS wild-type colorectal cancer [5-10]. We sought to screen for additional predictive biomarkers in a retrospective clinical series of 226 cetuximab-treated advanced CRC patients and to look for hypothesis-generating hints of efficacy or resistance. Despite homogeneous management of all patients in standardised HeCOG recommended therapies, the retrospective nature of the translational research is an inherent limitation. Moreover, in the absence of a control arm with no treatment it is impossible to speculate on the prognostic (impact on outcome irrespective of therapy) or predictive (impact on benefit from cetuximab therapy) effect of the biomarkers studied. Finally, the administration of cetuximab across several lines of therapy combined with various chemotherapeutic agents constitutes an additional layer of heterogeneity, masking possible interactions and confounders. Moreover, survival analysis will not be robust and even tumor response may be influenced by the chemotherapy backbone to which cetuximab was added. Finally, it would be ideal for all of the tissue samples to have been collected prior to cetuximab therapy, as some of the biomolecules studied may have changed by the time patients received 1st, 2nd or 3rd line therapy.

The incidence of observed KRAS, BRAF, NRAS, PIK3CA mutations are in line with those in the literature, ranging from 30-43% for KRAS, 0.5-7.2% for BRAF, 2-4% for NRAS, 5-17% for PIK3CA [8,16-19]. In univariate analysis, BRAF mutations were associated with an 8-fold increased risk of death, while KRAS mutations incurred a weaker, less than 2-fold increase in the risk of death only when codon 12 mutations were analysed separately. Similar to our findings, Souglakos et al. reported a markedly increased risk of death with a multivariate HR of 5.1 for BRAF mutations in cetuximab-treated CRC patients [20]. Moreover, Modest et al. recently reported median survival times of 23.5 months in KRAS wild-type, 18.9 months in KRAS codon 12-mutated and only 13 months in BRAF-mutated CRC patients on cetuximab-based first-line therapy [21]. Seemingly, BRAF mutation seems to carry especially adverse prognosis in patients who receive anti-EGFR therapeutic modulation.

We observed no prognostic effect of NRAS, PIK3CA or KRAS mutations outwith codon 12. Despite initial reports of significant negative correlation between PIK3CA mutations and response to anti-EGFR moAbs, series by Prenen et al. and Saridaki et al. failed to find such an association [10,18]. De Roock et al. suggested that only exon 20 PIK3CA mutations were associated with resistance to cetuximab therapies [8,22]. In our series, only 5 tumours with exon 20 PIK3CA mutations were detected. Regarding KRAS, the same group reported in a pooled dataset of 579 cetuximab-treated CRC patients that those with codon 13 KRAS mutant tumours (G13D) had better outcomes (HR for death 0.50) compared to patients with other KRAS mutant tumours [9]. Those findings stood in our Greek population cohort, as the median survival of our cetuximab treated patients seemed to de-escalate from 29–33 months for KRAS wild type to 28 months for the KRAS codon 13 mutated group, dropping to 19 months for the KRAS codon 12 mutated group. Although Peeters et al. recently failed to find a prognostic difference between codon 12 and codon 13 KRAS mutations in CRC patients managed with panitumumab in three randomised trials, another group from Germany did so for cetuximab [21,23,24]. KRAS codon 12 mutant CRC patients reached a median survival of only 18.9 months versus 23.5 months for KRAS wild type and 26.2 months for KRAS codon 13 mutant CRC patients on first line cetuximab+CAPIRI/CAPOX. Indeed in preclinical experiments, transforming potential, RAS/GTP activation, MAPK phosphorylation and tumour growth were markedly increased in the presence of KRAS codon 12 mutations, followed in decreasing order by codon 13, 61, 117 and 146 mutations [25,26]. Moreover, microarray profiling of multi-gene expression signatures associated with KRAS mutations revealed that codon 12 mutations were linked to a gene cluster distinct from all other KRAS mutations [25]. Further validation of these data is warranted in order to confirm or refute the hypothesis that specific molecular characteristics of KRAS mutations are determinants of cetuximab benefit.

We observed a strong correlation of increased tumoural mRNA of AREG, EREG with clinical benefit from cetuximab (>50% reduction in the risk of death), in keeping with data reported by four other groups [11,20,27,28]. Gene expession of the two ligands, which are collocalised on chromosome 4q13.3 and produced by tumour cells in autocrine fashion, was tightly correlated and occurred more often in KRAS and BRAF wild type tumours. The correlation of low EREG/AREG expression with KRAS or BRAF mutated status could be due to the constitutive activation of the RAS/RAF/MAPK pathway which makes activation of the EGFR pathway redundant biologically. Alternatively, it could be due to a negative feedback loop linking MAPK axis activation with suppression of the EGFR pathway.

Objective response to anti-EGFR therapy was correlated to continuous expression levels of AREG, EREG but only to categorical EREG mRNA distribution, intriguingly more strongly in KRAS mutated cases. The smooth nature of relationship between marker levels and outcome, resulting in lack of a universal cut-point applicable in all clinical situations has already been reported by Jacobs et al. [11]. In the presence of downstream activation of the EGFR/RAS/MAPK axis due to mutated KRAS effector, gene expression of AREG, EREG ligands would be biologically irrelevant for benefit from cetuximab. In our series this was the case for AREG, however tumour EREG mRNA retained predictive significance for survival both in KRAS wild-type as well as mutated cases, a counter-intuitive finding, further supported by the predictive significance of EREG for response, even in KRAS mutant patients. AREG and EREG are not biologically identical: AREG binds EGFR only, whereas EREG binds EGFR and HER4 and leads to a prolonged state of receptor activation [29]. Compared to AREG, tumour EREG mRNA expession was a stronger predictor of cetuximab benefit in KRAS wild type cases in three more series [13,20,27]. Consequently, we may speculate that even in the presence of KRAS mutations, cetuximab binding to EGFR prevents high levels of EREG from activating HER1/HER4 heterodimers and thus abrogates signalling pathways distinct from RAS/MAPK. In fact, Baker et al. reported HER4 gene expression as one of the genes significantly associated with clinical benefit in 144 cetuximab-treated CRC patients [28]. However, our findings should be interpreted with caution as the number of EREG-high, KRAS-mutant cases analysed was low (n=10) and random effects cannot be excluded.

In our cohort, the survival of cetuximab-treated patients with KRAS wild type, AREG-low CRC (median 15 months) was as poor as the survival of patients with KRAS mutant tumours (17–22 months). If this finding is confirmed in independent series, AREG expression in KRAS wild type cases would emerge as a robust biomarker of cetuximab efficacy. Baker et al. suggested the use of a 4-gene score (AREG, EREG, DUSP6, SLC26A3) for the prediction of anti-EGFR treatment benefit in KRAS wild type CRC [28]. In the presence of low AREG and thus inactive EGFR pathway, cetuximab binding to HER dimers might elicit a paradoxical «pro-survival» cellular response via MAPK-dependent and MAPK-independent pathways, as suggested by Oliveras-Ferraros et al. [30]. Finally, the markedly increased risk of death (8-fold) of high AREG mRNA in KRAS mutant versus KRAS wild-type CRC patients in multivariate analysis could be explained if AREG is viewed as a protein regulated by KRAS-dependent transcription factors: the former group includes tumours bearing KRAS mutations with a markedly pro-survival, proliferative activating effect, whereas the latter group includes KRAS wild type tumours addicted to active EGFR signalling that is amenable to abrogation by cetuximab.

## Conclusions

Our study confirms published data on the prognostic/predictive significance of BRAF, KRAS mutations and on the tumour expression of AREG, EREG mRNA in cetuximab-treated patients with colorectal cancer. Moreover, we present data suggesting a) a differential impact of non-codon 12 KRAS mutations on outcome of cetuximab-treated patients of Greek ethnic origin, b) a KRAS mutation-independent predictive significance of EREG mRNA expression, c) a strong predictive value of AREG mRNA expression in KRAS wild type patients. These findings warrant independent validation.

## Competing interests

The authors declare that they have no competing interests.

## Authors’ contributions

GP: Conceived of the study and drafted the manuscript. VK: Carried out the molecular genetic studies. WDR, ST: Carried out the mutational testing. GK: Performed the statistical analysis. DT: Reviewed histologically the tumour samples. PP, TM, DP, IX, JS, GK, ACT, ER, EG, DB, IE, IB, DP, NP, GF: Participated in the design and coordination of the study. All authors read and approved the final manuscript.

## Pre-publication history

The pre-publication history for this paper can be accessed here:

http://www.biomedcentral.com/1471-2407/13/49/prepub

## References

[B1] Jemal A Siegel R Ward E Cancer Statistics, 2009 CA Cancer J Clin 2009 59 225 249 10.3322/caac.20006 19474385

[B2] Siena S Sartore-Bianchi A Di Nicolantonio F Biomarkers Predicting Clinical Outcome of Epidermal Growth Factor Receptor-Targeted Therapy in Metastatic Colorectal Cancer J Natl Cancer Inst 2009 101 1308 1324 10.1093/jnci/djp280 19738166 PMC2758310

[B3] Linardou H Briasoulis E Dahabreh IJ All about KRAS for clinical oncology practice: gene profile, clinical implications and laboratory recommendations for somatic mutational testing in colorectal cancer Cancer Treat Rev 2011 37 221 233 10.1016/j.ctrv.2010.07.008 20817364

[B4] Scheffzek K Ahmadian MR Kabsch W The Ras-RasGAP complex: structural basis for GTPase activation and its loss in oncogenic Ras mutants Science 1997 277 333 338 10.1126/science.277.5324.333 9219684

[B5] Amado RG Wolf M Peeters M Wild-type KRAS is required for panitumumab efficacy in patients with metastatic colorectal cancer J Clin Oncol 2008 26 1626 1634 10.1200/JCO.2007.14.7116 18316791

[B6] Van Cutsem E Kohne CH Hitre E Cetuximab and chemotherapy as initial treatment for metastatic colorectal cancer N Engl J Med 2009 360 1408 1417 10.1056/NEJMoa0805019 19339720

[B7] Van Cutsem E Kohne CH Lang I Cetuximab plus irinotecan, fluorouracil, and leucovorin as first-line treatment for metastatic colorectal cancer: updated analysis of overall survival according to tumor KRAS and BRAF mutation status J Clin Oncol 2011 29 2011 2019 10.1200/JCO.2010.33.5091 21502544

[B8] De Roock W Claes B Bernasconi D Effects of KRAS, BRAF, NRAS, and PIK3CA mutations on the efficacy of cetuximab plus chemotherapy in chemotherapy-refractory metastatic colorectal cancer: a retrospective consortium analysis Lancet Oncol 2010 11 753 762 10.1016/S1470-2045(10)70130-3 20619739

[B9] De Roock W Jonker DJ Di Nicolantonio F Association of KRAS p.G13D mutation with outcome in patients with chemotherapy-refractory metastatic colorectal cancer treated with cetuximab JAMA 2010 304 1812 1820 10.1001/jama.2010.1535 20978259

[B10] De Roock W Piessevaux H De Schutter J KRAS wild-type state predicts survival and is associated to early radiological response in metastatic colorectal cancer treated with cetuximab Ann Oncol 2008 19 508 515 17998284 10.1093/annonc/mdm496

[B11] Jacobs B De Roock W Piessevaux H Amphiregulin and epiregulin mRNA expression in primary tumors predicts outcome in metastatic colorectal cancer treated with cetuximab J Clin Oncol 2009 27 5068 5074 10.1200/JCO.2008.21.3744 19738126

[B12] Sanchez-Navarro I Gamez-Pozo A Gonzalez-Baron M Comparison of gene expression profiling by reverse transcription quantitative PCR between fresh frozen and formalin-fixed, paraffin-embedded breast cancer tissues Biotechniques 2010 48 389 397 10.2144/000113388 20569212

[B13] Koutras AK Kalogeras KT Dimopoulos MA Evaluation of the prognostic and predictive value of HER family mRNA expression in high-risk early breast cancer: a Hellenic Cooperative Oncology Group (HeCOG) study Br J Cancer 2008 99 1775 1785 10.1038/sj.bjc.6604769 18985033 PMC2600696

[B14] Nishino M Jagannathan JP Ramaiya NH Van den Abbeele AD Revised RECIST guideline version 1.1: What oncologists want to know and what radiologists need to know AJR Am J Roentgenol 2010 195 281 289 10.2214/AJR.09.4110 20651182

[B15] Miller R Siegmund D Maximally Selected Chi-Square Statistics Biometrics 1982 38 1011 1016 10.2307/2529881

[B16] Sartore-Bianchi A Martini M Molinari F PIK3CA Mutations in Colorectal Cancer Are Associated with Clinical Resistance to EGFR-Targeted Monoclonal Antibodies Cancer Res 2009 69 1851 1857 10.1158/0008-5472.CAN-08-2466 19223544

[B17] Siena S Sartore-Bianchi A Di Nicolantonio F Balfour J Bardelli A Biomarkers predicting clinical outcome of epidermal growth factor receptor-targeted therapy in metastatic colorectal cancer J Natl Cancer Inst 2009 101 19 1308 1324 10.1093/jnci/djp280 19738166 PMC2758310

[B18] Prenen H De Schutter J Jacobs B PIK3CA mutations are not a major determinant of resistance to the epidermal growth factor receptor inhibitor cetuximab in metastatic colorectal cancer Clin Cancer Res 2009 15 3184 3188 10.1158/1078-0432.CCR-08-2961 19366826

[B19] Perrone F Lampis A Orsenigo M PI3KCA/PTEN deregulation contributes to impaired responses to cetuximab in metastatic colorectal cancer patients Ann Oncol 2009 20 84 90 18669866 10.1093/annonc/mdn541

[B20] Saridaki Z Tzardi M Papadaki C Impact of KRAS, BRAF, PIK3CA mutations, PTEN, AREG, EREG expression and skin rash in >/= 2 line cetuximab-based therapy of colorectal cancer patients PLoS One 2011 6 e15980 10.1371/journal.pone.0015980 21283802 PMC3024325

[B21] Modest DP Jung A Moosmann N The influence of KRAS and BRAF mutations on the efficacy of cetuximab-based first-line therapy of metastatic colorectal cancer: An analysis of the AIO KRK-0104-trial Int J Cancer 2012 131 4 980 986 10.1002/ijc.26467 21960311

[B22] De Roock W De Vriendt V Normanno N KRAS, BRAF, PIK3CA, and PTEN mutations: implications for targeted therapies in metastatic colorectal cancer Lancet Oncol 2011 12 594 603 10.1016/S1470-2045(10)70209-6 21163703

[B23] Douillard JY Siena S Cassidy J Randomized, phase III trial of panitumumab with infusional fluorouracil, leucovorin, and oxaliplatin (FOLFOX4) versus FOLFOX4 alone as first-line treatment in patients with previously untreated metastatic colorectal cancer: the PRIME study J Clin Oncol 2010 28 4697 4705 10.1200/JCO.2009.27.4860 20921465

[B24] Peeters M Evaluation of Individual Codon 12 and 13 Mutant (MT) KRAS Alleles as Prognostic and Predictive Biomarkers of Response to Panitumumab (pmab) in Patients with Metastatic Colorectal Cancer ECCO 2011 2011 20 10.1200/JCO.2012.45.1492 23182985

[B25] Smith G Bounds R Wolf H Activating K-Ras mutations outwith ‘hotspot’ codons in sporadic colorectal tumours - implications for personalised cancer medicine Br J Cancer 2010 102 693 703 10.1038/sj.bjc.6605534 20147967 PMC2837563

[B26] Janakiraman M Vakiani E Zeng Z Genomic and biological characterization of exon 4 KRAS mutations in human cancer Cancer Res 2010 70 5901 5911 10.1158/0008-5472.CAN-10-0192 20570890 PMC2943514

[B27] Khambata-Ford S Garrett CR Meropol NJ Expression of epiregulin and amphiregulin and K-ras mutation status predict disease control in metastatic colorectal cancer patients treated with cetuximab J Clin Oncol 2007 25 3230 3237 10.1200/JCO.2006.10.5437 17664471

[B28] Baker JB Dutta D Watson D Tumour gene expression predicts response to cetuximab in patients with KRAS wild-type metastatic colorectal cancer Br J Cancer 2011 104 488 495 10.1038/sj.bjc.6606054 21206494 PMC3049558

[B29] Shelly M Pinkas-Kramarski R Guarino BC Epiregulin is a potent pan-ErbB ligand that preferentially activates heterodimeric receptor complexes J Biol Chem 1998 273 10496 10505 10.1074/jbc.273.17.10496 9553109

[B30] Oliveras-Ferraros C Vall-Llovera AM Salip DC Evolution of the predictive markers amphiregulin and epiregulin mRNAs during long-term cetuximab treatment of KRAS wild-type tumor cells Invest New Drugs 2012 30 846 852 10.1007/s10637-010-9612-2 21161326

